# Identifying prospective temperament predictors of callous-unemotional traits using machine learning

**DOI:** 10.1007/s00787-026-03012-8

**Published:** 2026-03-19

**Authors:** Alexis Broussard, Sarah C. Vogel, Patrick K. Goh, Emily R. Perkins, Yael Paz, Nicole Huth, Anthony J. Rosellini, William R. Mills-Koonce, Michael T. Willoughby, Rebecca Waller, Nicholas J. Wagner

**Affiliations:** 1 Department of Psychology, University of Pennsylvania, Philadelphia, Pennsylvania US; 2 Department of Brain and Psychological Science, Boston University, Boston, Massachusetts US; 3 Department of Psychology, University of Massachusetts, Boston, Massachusetts US; 4 Department of Epidemiology, Boston University School of Public Health, Boston, Massachusetts US; 5 Department of Psychology, University of Hawaiʻi at Mānoa, Honolulu, Hawaii US; 6 Development and Family Studies, University of North Carolina, Greensboro, North Carolina US; 7 Research Triangle Institute (RTI International), Research Triangle Park, North Carolina US

**Keywords:** Callous-unemotional traits, Fear, Affiliation, Conduct disorder, Machine learning

## Abstract

Children with callous-unemotional (CU) traits (i.e., low guilt, restricted empathy) are at high risk for disruptive behavior disorders (DBD) across development. The Sensitivity to Threat and Affiliative Reward (STAR) model posits that low fear and low affiliation (i.e., disrupted social bonding motivation) are temperament dimensions that increase risk for CU traits. However, prior tests of the STAR model are limited by the lack of prospective longitudinal studies and reliance on short-term, single-timepoint, single-measure assessments. We applied machine learning to repeated observational measures of temperament across infancy and preschool to test whether STAR model features (i.e., fear, affiliation), alongside other temperament constructs (e.g., frustration, activity, persistence), predicted CU traits at age 7. Data were from the Family Life Project (FLP), a birth cohort study (*N =* 1,292) that oversampled families with low household incomes. We used random forest models to predict CU traits and conduct disorder (CD) symptoms at age 7 using 39 features derived from observed temperament measures assessed at ages 6, 15, 24, 35, and 48 months. Models explained only 2% of the variance in CU traits at age 7, with behavioral observations of positive affect and persistence at 48 months among the strongest predictors. Although temperament measures relevant to affiliation were modestly predictive of CU traits, findings overall provide weak evidence for the STAR model.

**Supplementary Information:**

The online version contains supplementary material available at 10.1007/s00787-026-03012-8.

Callous-unemotional (CU) traits (low guilt, lack of empathy) predict very high risk for conduct disorder (CD), antisocial behavior, and psychopathic traits [1], as well as poor educational attainment and difficulties with social relationships [2]. Children with CU traits have worse treatment outcomes following standard interventions for CD, ending treatment with greater symptom severity than those with CD alone [3]. CD generates vast societal and economic costs related to greater utilization of health, educational, welfare, and criminal justice services [4]. Thus, enhanced knowledge of how CU traits develop can improve our ability to identify children at risk for CD and inform more effective personalized interventions to mitigate the deleterious consequences of antisocial behavior.

The Sensitivity to Threat and Affiliative Reward (STAR) model proposes that low fear and low affiliation are risk factors for CU traits [5]. Fear refers to behavioral, cognitive, and emotional responses that enhance safety or behavioral change in response to real or perceived environmental threats [6]. Low fear is thought to increase risk for CU traits by disrupting children’s ability to learn that their behavior is harmful based on social (e.g., distress, anger) and non-social (e.g., punishment, danger) cues, impairing the development of conscience and rule-governed behavior [7]. Affiliation is the motivation for relationships arising from the rewarding nature of positive social bonding experiences [8]. Low affiliation is thought to increase risk for CU traits by disrupting children’s initiation and enjoyment of social interactions, undermining the development of caring, empathic, and prosocial behaviors [5].

Supporting evidence for the STAR model comes from studies that have linked low fear in early childhood to later CU traits, with fear operationalized through observed measures of reduced orientation to novel people or situations [9, 10], and reduced responsiveness to fear-inducing stimuli [11], as well as report measures [12]. Similarly, low affiliation in early childhood has been linked to increases in CU traits, with low affiliation assessed using parent report [13] and observations of low eye contact [14], fewer affectionate signals [15], reduced social imitation [16], and restricted positive affect in social situations [17]. Finally, a handful of studies have demonstrated that the combination of both low fear and low affiliation (i.e., indexed by a statistical interaction) explains unique variance in CU traits across development [12, 13].

Extant literature supports low fear and low affiliation as precursors to the development of CU traits, with longitudinal studies showing that low affiliation and low fear are temporally related to increases in CU traits [13, 16–18]. For example, Domínguez-Álvarez et al. (2021) found that parent-reported fearlessness and low affiliation were related to increases in parent-reported CU traits one year later in a community sample of Spanish preschoolers [13]. Additionally, Waller et al. (2016) found that early observational measures of low fear and low affiliation at 18 months were related to higher adoptive mother-reported CU traits at 27 months [18]. Both longitudinal and cross-sectional studies also indicate a stronger unidirectional path from low affiliation to CU traits, providing evidence that these temperament dimensions precede increases in CU traits, rather than the reverse [19, 20].

However, most studies that have tested the STAR model have focused on the preschool period, used brief follow-up periods (i.e., 1 to 2 years only), and relied on report measures. We particularly need knowledge of CU traits during middle childhood, a period when important developmental changes occur that shape persistence or desistance from long-term antisocial behavior [21]. Moreover, no studies have tested whether the STAR dimensions predict CU traits better than other established temperament risk factors for CD, including irritability or frustration [22]. Similarly, studies have not compared whether the STAR dimensions show similar predictive power in relation to CU traits versus CD or attention deficit hyperactivity disorder (ADHD), which has implications for identifying different externalizing subtypes and tailoring early preventative interventions [23]. Finally, prior studies differ based on *when* and *how* they measure the STAR dimensions, which has been offered as an explanation for some mixed findings to date [5]. For example, some studies have failed to find direct links between low fear and CU traits [11] or report that greater fear relates to higher CU traits, which may be a function of the time point or measure used to assess fear [24, 25].

To address these limitations, we need systematic, objective, and longitudinal study designs that leverage repeated, diverse measures of the STAR model and other temperament constructs across infancy and early childhood to establish *which dimensions* at *which ages* most strongly predict CU traits later in childhood. One approach to addressing this question is through traditional regression-based analysis, which evaluates the unique contribution to the variance in CU traits or CD by different temperament features at various ages. However, regression methods can limit model complexity in terms of the number of predictors that can be simultaneously evaluated, especially when predictors are correlated with one another, risk overfitting the data, and offer limited replicability. Regression can also fail to account for interactions and non-linear associations between predictors and outcomes. To address these issues, data-driven machine learning (ML) approaches appropriate for prediction analyses are needed [26], though studies have yet to apply ML to test the STAR model.

Supervised ML approaches, such as random forest, offer an alternative approach to address these limitations and provide several advantages over traditional regression models. First, random forest methods are better equipped to handle highly multidimensional data, including accommodating a large set of predictors whose relative importance can be compared to better pinpoint which variables are most strongly associated with the outcome of interest [26]. Second, random forest ML utilizes ensemble learning to address overfitting and multicollinearity [27, 28]. That is, by aggregating predictions from numerous decision trees, each trained on a random subset of data and predictors, the model is better able to capture stable underlying patterns within the data, leading to better performance on unseen data [27, 28]. Finally, random forest models can identify and model complex interactions and nonlinear relationships because within each decision tree, the deciding parameter at each split depends on parameters used in preceding splits [29, 30]. Thus, contingencies between predictors (i.e., interactions) and nonlinear patterns between predictors and outcomes can be automatically modeled, allowing the model to adaptively learn from the data and produce more accurate predictions [29, 30].

Thus, in the current study, we applied ML methods to prospective longitudinal data, with repeated observational assessments of STAR and other temperament dimensions across infancy and preschool age, to identify predictors of CU traits and CD symptoms at age 7. We used a random forest algorithm because of its superior accuracy and model fit when applied to multidimensional data [31], enhanced generalizability in detecting non-linear relationships, and ability to identify the variables that contributed most strongly to the prediction [26]. By leveraging random forest methods, our aim was to provide a rigorous test of the STAR model, deriving generalizable findings and determining the relative predictive influence of STAR and other temperament dimensions on risk for CU traits and CD across multiple time points. We also compared the findings using a more traditional regression-based approach where we entered summary scores across temperament dimensions as independent variables and evaluated specificity in relation to CU traits, CD symptoms, and ADHD symptoms.

## Method

### Sample

Data were from the Family Life Project (FLP), a birth cohort study of *N =* 1,292 children and families from two rural, high-poverty regions in the United States (three counties from Eastern North Carolina and three counties from Central Pennsylvania; see [32] for details). Recruitment spanned September 2003 to September 2004, oversampling for African American families in North Carolina. Table 1 presents demographic characteristics of the sample at two months, when families initially enrolled in the study, as well as descriptive information for key study variables. We excluded participants who were missing continuous scores on CU traits or CD symptoms at age 7 or more than 33% of temperament predictors. The current study included *n =* 978 (50% female; 44% Black and 3% Latina/e/o/x) (Table 1). Excluded participants did not differ from included participants based on race, sex, site, income-to-needs ratio, CU traits, CD symptoms, or ADHD symptoms (Table S1).

**Table 1:** Sample Demographics and Descriptive Information on Key Study Variables

	Age 7: (*n* = 978)		
	*N*	%	
Sex			
Male	489	50%	
Female	489	50%	
Race^a^			
Black	426	44%	
White	547	56%	
Ethnicity^b^			
Spanish/Hispanic/Latino	25	3%	
Non-Spanish/Hispanic/Latino	951	97%	
State			
North Carolina	579	59%	
Pennsylvania	399	41%	
	***M***	***SD***	***Range***
Income-to-needs ratio^c^	1.90	1.67	0–16.49
Outcomes			
CU traits	16.61	9.77	0–53
CD symptoms	0.23	0.90	0–9
ADHD symptoms	15.54	11.67	0–57

^**a**^ Five participants were American Indian. ^b^ Two participants did not have ethnicity data available. ^c^ 92 participants did not have income-to-needs ratio data available

### Data collection procedures

Families completed two home visits when children were 6, 24, and 35 months old and one home visit at 15 and 48 months old. Biological mothers made up 96–99% of primary caregivers at each age, so we focused on including data collected from mothers [32]. During visits, primary caregivers completed questionnaires, two experimenters completed ratings of child temperament based on their observations, and coders later rated videotaped structured tasks completed at home [32].

## Measures

A summary of each measure and timepoint for all 39 temperament predictors is presented in Table 2. Intercorrelations between ratings from the two visits at one timepoint are presented in Table S2. The observed temperament scales were correlated across visits within timepoints (*r*=.35–0.67, *p*s<0.001), so we created mean composites averaged across experimenters and visits within timepoints for each construct (see Table S2 and descriptions below).

**Table 2:** Construct- and Age-Specific Predictors

Constructs		Ages (months)				
	Predictors	6	15	24	35	48
Fear	Fear reactivity	**+**	**+**	**+**		
	Fear to new/strange	**●**	**●**	**●**	**■**	**■**
Affiliation	Responsiveness to persons	**●**	**●**	**●**		
	Responsiveness to examiner	**●**	**●**	**●**	**■**	**■**
	Responsiveness to caregiver	**●**	**●**	**●**		
	Happiness/Positive affect	**●**	**●**	**●**	**■**	**■**
Self-Regulation	Attention	**●**	**●**	**●**		
	Persistence				**■**	**■**
Activity	Gross movement/Activity	**●**	**●**	**●**	**■**	**■**
Negative Emotionality	Irritability	**●**	**●**	**●**		
	Frustration				**■**	**■**

We summarize the five higher order temperament constructs (i.e., fear, affiliation, self-regulation, activity, negative emotionality), their corresponding predictors, and the ages at which predictors were assessed. IBR = Infant Behavior Record. OCTS = Observation of Child Temperament Scale. + Mask Task, ● IBR, ■ OCTS

## Fear

Fear reactivity was assessed using a scary mask task at ages 6, 15, and 24 months [33]. Children saw four increasingly frightening masks worn by an experimenter. Children’s responses were videotaped and analyzed with microanalytic coding using the Better Coding Approach software, with fear reactivity coded as low, moderate, or high [34]. Consistent with prior studies, we summed fear reactivity (by the second) across the task and divided scores by task duration to create a proportion score for fear reactivity [35]. Coders achieved inter-rater reliability based on double coding of 15% of videos (6 months, *kappa=*0.94; 15 months, *kappa*=0.89; 24 months, *kappa=*0.90).

We assessed children’s fearfulness to new or strange persons, objects, or situations using experimenter ratings from an adapted version of the Infant Behavior Record (IBR) at 6, 15, and 24 months [36] and the Observed Child Temperament Scale (OCTS) at 35 and 48 months [36]. Ratings were on a 9-point scale (1 = *no evidence of fear*,* caution*,* or inhibited action*, 5 = *moderately affected for the first third of the visit*, 9 = *strong indication of fear*). Both experimenters completed ratings immediately following each home visit using Blaise software, and their ratings were averaged. The IBR has been used extensively in its original form [37, 38] and the OCTS has been used previously as a valid measure of infant temperament [39]. Inter-rater intraclass correlations for all items demonstrated moderate to good reliability at each time point (ICC=0.53-0.81; see Table S3). Further, confirmatory factor analysis (CFA) established that the fear items loaded onto a unidimensional fear factor (CFI=0.96, RMSEA=0.04; Table S4).

## Affiliation

Responsiveness to persons, examiner, and caregiver were measured using the IBR and the OCTS. The responsiveness to persons item assessed children’s overall responsiveness to all persons present at the home visit and was measured at 6, 15, and 24 months. Ratings were on a 9-point scale (1 = *behavior towards persons is not different from behavior towards objects*, 5 = *responds to social approach and persons present less than half the time*, 9 = *behavior is continuously affected by awareness of persons present*). The responsiveness to examiner and caregiver items were rated on a 5-point scale (e.g., 1 = *avoiding or withdrawn*, 3 = *accepting*, 5 = *inviting*,* initiating*,* demanding*). The responsiveness to examiner item was measured at 6, 15, 24, 35, and 48 months and the responsiveness to caregiver item was measured at 6, 15, and 24 months. Positive affect was measured using the happiness item from the IBR (6, 15, and 24 months) and the positive affect item from the OCTS (35 and 48 months). Items were rated on a 9-point scale (1 = *unhappy throughout visit*, 5 = *moderately happy or contented; may become upset*,* but recovers fairly easily*; 9 = *radiates happiness; nothing is upsetting; animated*). Inter-rater intraclass correlations for all items demonstrated moderate to good reliability at each time point (ICC=0.50-0.83; see Table S3). Further, CFA established that the affiliation items loaded onto a unidimensional affiliation factor (CFI=0.94, RMSEA=0.06; Table S4).

## Activity

Activity was assessed using the gross movement item from the IBR (6, 15, and 24 months) and the activity level item from the OCTS (35 and 48 months). Items were rated on a 9-point scale (1 = *stays quietly in one place*,* with practically no self-initiated movement*; 5 = *moderate activity; enters play with freedom of action*; 9 = *hyperactive; difficulty during sedentary tests*). Inter-rater intraclass correlations for all items demonstrated moderate to good reliability at each time point (ICC=0.67-0.74; see Table S3). Further, CFA established that all activity items loaded onto a unidimensional activity factor (CFI=0.96, RMSEA=0.07; Table S4).

### Self-regulation

Attention was measured at 6, 15, and 24 months using three items from the IBR: (1) responsiveness to objects, (2) tendency to persist in attending to any one object, person, or activity apart from attaining a goal, and (3) behavior constancy in adequately responding to demands of the home visit. Items were rated on a 9-point scale (e.g., 1 = *fleeting attention*, 5 = moderated attention, 9 = long-continued absorption). The three items were summed to create an overarching attention scale (Table S2) [36]. Persistence was measured at 35 and 48 months using the task persistence item from the OCTS. Items were rated on a 9-point scale (1 = *does not stay on task*, 5 = *persists with tasks that interest him/her*; 9 = *shows high task persistence*). Inter-rater intraclass correlations for all items demonstrated moderate to good reliability at each time point (ICC=0.71-0.82; see Table S3). Further, CFA established that the self-regulation items loaded onto a unidimensional self-regulation factor (CFI=0.97, RMSEA=0.07; Table S4).

## Negative emotionality

Irritability was measured at 6, 15, and 24 months using one item from the IBR rated on a 9-point scale (1 = *no irritability*, 5 = *irritability to aversive and non-aversive stimulation leads to high intensity crying*,* but with consoling returns to lower states*; 9 = *irritable to all degrees of stimulation*) [29]. Frustration was measured at 35 and 48 months using one item from the OCTS assessing the degree of anger, including during frustration tasks. This item was rated on a 9-point scale (1 = *no evidence of frustration even during tasks designed to frustrate*; 5 = *moderate frustration but regulates well*; 9 = *easily frustrated*,* uncontrolled anger*). Inter-rater intraclass correlations for all items demonstrated moderate to good reliability at each time point (ICC=0.73-0.80; see Table S3). Further, CFA established that all negative emotionality items loaded onto a unidimensional negative emotionality factor (CFI=0.97, RMSEA=0.05; Table S4).

## Outcome variables

CU traits were assessed at age 7 using caregiver report on the Inventory of Callous-Unemotional Traits (ICU) [40], an established 24-item measure assessing callousness (e.g., shows no remorse), uncaring (e.g., unconcerned about schoolwork), and unemotionality (e.g., does not show emotions), with items rated on a 4-point Likert scale (0 = *not at all true*, 1 = *somewhat true*, 2 = *very true*, 3 = *definitely true*). Consistent with prior research [41], we used a summed total score across all 24 items to index CU traits (*α*=0.71; Table S5).

CD symptoms were assessed at age 7 using caregiver reports on 9 items assessing CD from the 45-item Disruptive Behavior Disorder Rating Scale (DBDRS) [42], an established DSM-based rating scale, with items rated on a four-point scale (0 = *not at all* to 3 = *very much*; e.g., bullies, threatens, or intimidates others). We computed total scores to index CD symptom severity (*α*=0.92; Table S5).

ADHD was assessed at age 7 using caregiver reports on 18 DSM-IV ADHD symptoms (e.g., easily distracted, difficulty awaiting turn). Items were rated on a 4-point Likert-like scale (0 = not at all, 1 = just a little, 2 = pretty much, and 3 = very much). Consistent with prior work [43], we computed a mean ADHD symptom score across items (α = 0.95; Table S3).

### Analytic strategy

Analyses were conducted in R [44]. Prior ML work suggests removing predictors with over 50% missingness [45]. No features met this threshold, so all were retained in the dataset (i.e., 39 features). Missing values for the 39 features (3% of the dataset) were imputed using the missForest package, which implements a random forest single imputation algorithm for missing data [46]. All predictors were treated as continuous and standardized. We used the caret package in R [47] to train the Random Forest (RF) algorithm, a non-parametric ensemble learning method that predicts outcome variables by aggregating the results of numerous decision trees. The RF algorithm emphasizes feature selection, evaluating whether certain predictors are more important than others without assuming linear relationships between predictor and outcome variables. To increase generalizability, we randomly split participants into training (75% of sample; *n* = 734) and testing (25% of sample; *n* = 244) samples. Next, we applied 5-fold repeated cross-validation to the training sample to tune the random forest models via the mtry hyperparameter. To do so, a subsample was drawn from the training sample through bootstrapping procedures, with 80% of this subsample (i.e., four of the five “folds”) used to train a tree and 20% (i.e., the last “fold”) used to test the tree. This process was repeated 5 times in total (each fold served as the test sample for a tree once). Within each tree, 38 randomly selected predictors were tested at each “split” to prevent overfitting. The entire cross-validation was iterated 100 times in total.

Next, we used recursive feature elimination (RFE) to find the most efficient number of features for predicting outcomes (i.e., CU traits and CD symptoms). RFE builds a model using all features, calculates the importance of each feature, rank-orders them, and removes those with the least importance based on model performance metrics (e.g., lowest Root Mean Squared Error, RMSE; highest R-squared, R^2^). The optimal model minimized the discrepancy between predicted and observed outcome scores (RMSE) and maximized the proportion of variance in outcomes explained (R^2^). The RF regression was repeated to create a final model including only the RFE-selected set of predictors. We then tested how well our algorithm predicted outcomes (i.e., *R*^2^) in the testing sample. Finally, we generated variable importance values reflecting top features from the RFE model and full model (all 39 features). Variable importance reflects how much the random forest model’s prediction accuracy decreases when a predictor’s values are randomly shuffled [48, 49]. If shuffling a variable largely reduces accuracy, that variable is considered an important contributor to the model’s predictive performance [48, 49]. If there is little or no change, the variable is considered a less important contributor [48, 49]. Directionality of associations between predictors and outcomes were evaluated by examining pairwise correlations.

In *post hoc* analysis, we tested specificity in the prediction of CU traits versus broader externalizing psychopathology symptoms. First, we computed within-domain summary scores across all 39 features (e.g., mean of all *z-*scored affiliation, activity, negative emotionality, fear, or self-regulation items) and then entered each domain score as independent variables in a regression model predicting CU traits, CD, or ADHD symptoms. Confirmatory factor analyses established that within-domain features mapped onto domain-level constructs (see Table S4). Second, for comparison purposes, we ran an RF model predicting *residualized* CU traits scores at age 7, regressing out conduct disorder symptoms, and generated variable importance values reflecting top features.

## Results

We report model performance metrics from the testing set only to quantify generalizability [26]. We focused our interpretation on the top 10 most important predictors across models for parsimony (i.e., ≈ 25%). Output from the training data, including RF models, intercorrelations between all variables, and variable importance values, are in the Supplementary Results.

### Which temperament measures, at which ages, best predict CU traits at age 7?

**Fig. 1:**
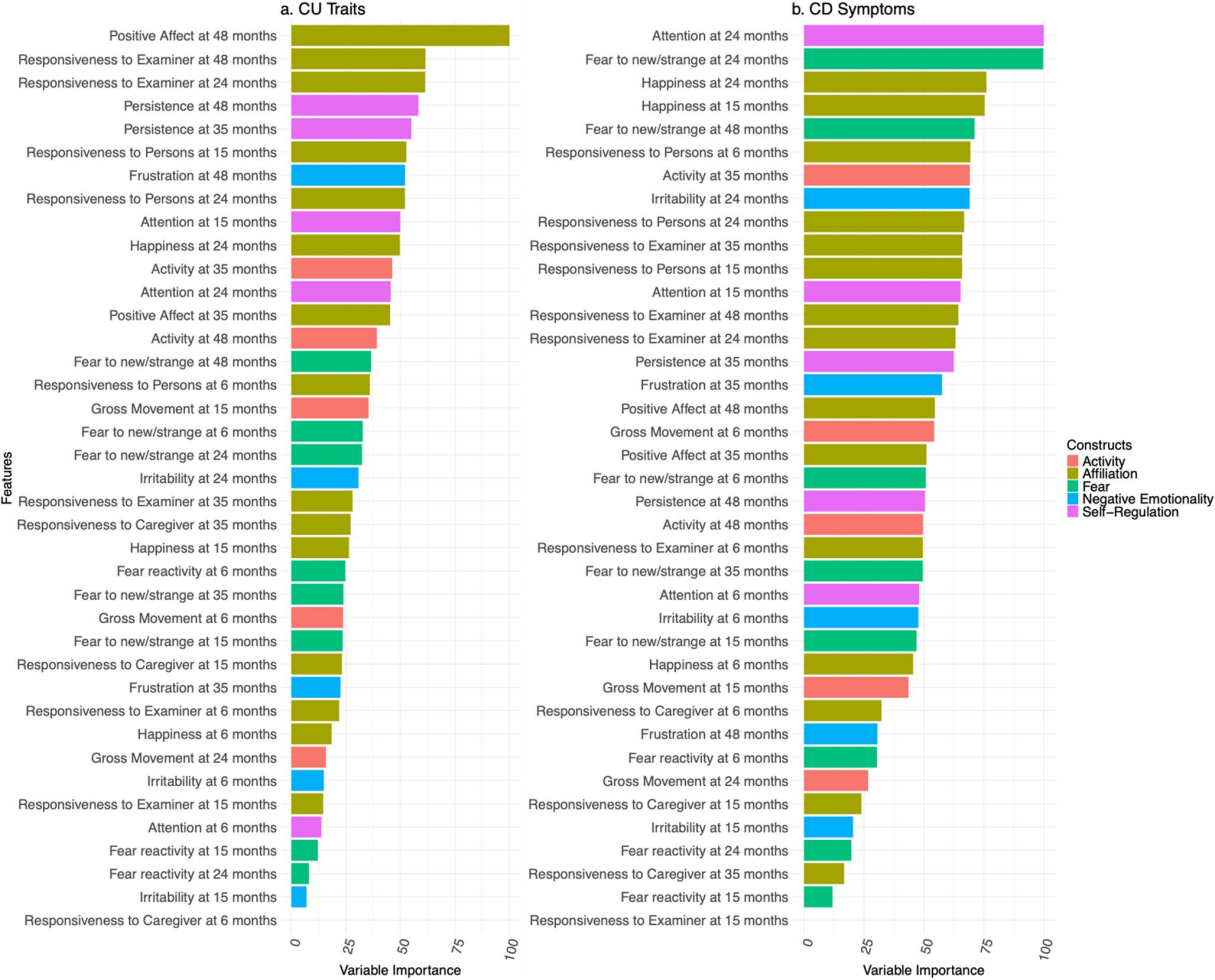
Variable Importance Plots from Full Random Forest Models. Variable importance scores from the first random forest model (including all 39 features) in the training sample are shown. Features are depicted on the y-axis and importance scores on the x-axis. Higher scores indicate greater importance

**Fig. 2:**
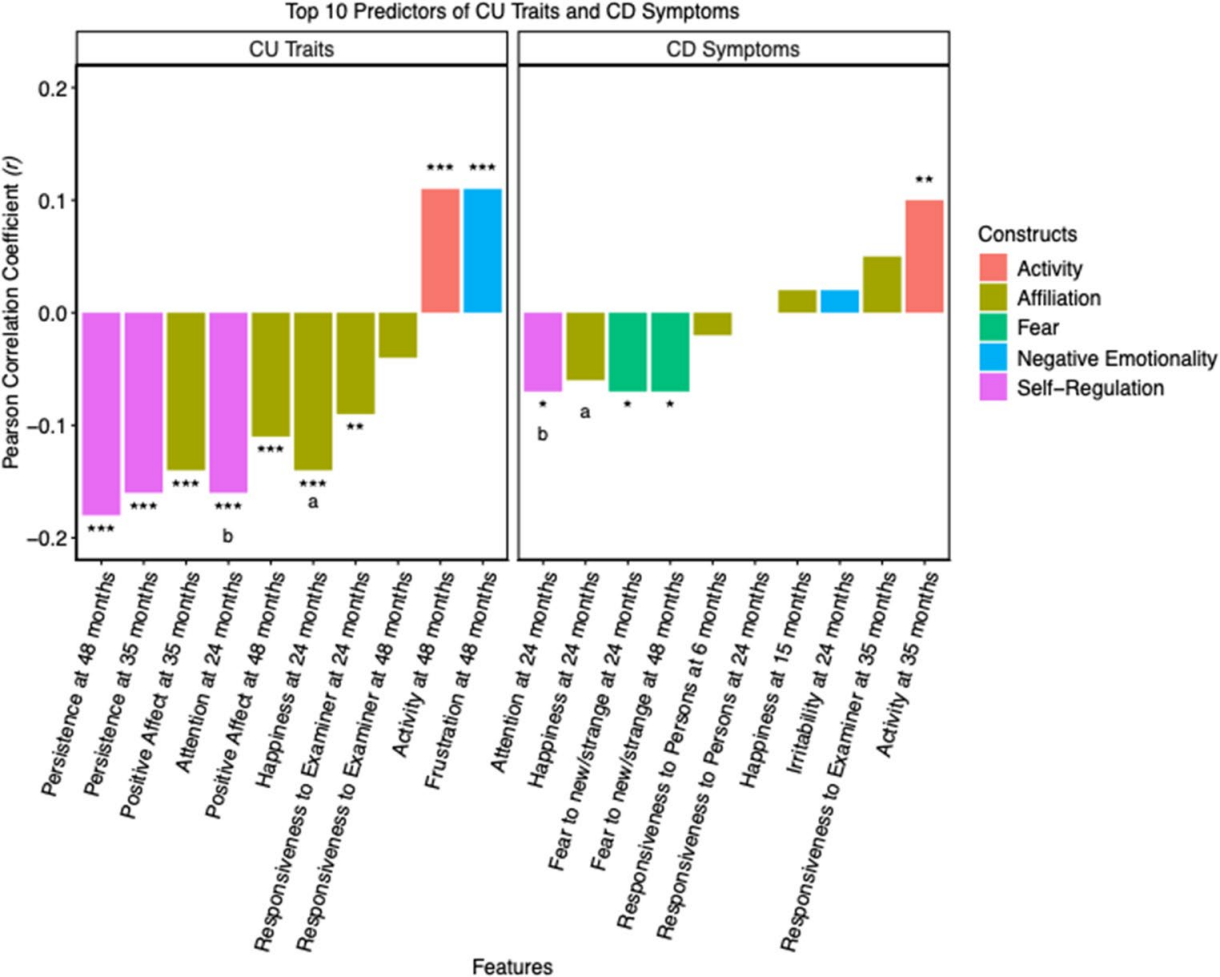
Summary of Top 10 Predictors of CU Traits and CD Symptoms. Pearson’s correlations (r) between the top 10 predictors of CU traits (left) and CD symptoms (right) are shown. Top predictors were identified by pulling features that emerged within the top 15 variables across importance lists from the full and RFE random forest models. Shared predictors of both CU traits and CD symptoms are indicated by a (Happiness at 24 months) and b (Attention at 24 months). Responsiveness to persons at 24 months was not linearly correlated with CD symptoms (r = .00, p > 0.05), so its correlation coefficient does not appear on the plot. ***p < .001, **p < .01, *p < .05

Using the full set of 39 predictors, the random forest procedure selected a model with three predictors at each split, explaining 2% of the variance in CU traits (*R*^*2*^=0.02, *RMSE* = 9.20). RFE procedures did not significantly improve the explained variance in CU traits (Tables S6). Thus, we retained the full set of 39 original predictors. Variable importance rankings from the full model are shown in Fig. 1a. The top 10 predictors across the full and RFE models are as follows, with correlation coefficients shown to index directionality: lower persistence at 48 months (*r*=-.18, *p*<.001), lower persistence at 35 months (*r*=-.16, *p*<.001), lower positive affect at 35 months (*r*=-.14, *p*<.001), lower attention at 24 months (*r*=-.16, *p*<.001), lower positive affect at 48 months (*r*=-.11, *p*<.001), lower happiness at 24 months (*r*=-.14, *p*<.001), lower responsiveness to examiner at 24 months (*r*=-.09, *p*<.01), lower responsiveness to examiner at 48 months (*r*=-.04, *p*=.19), higher activity at 48 months (*r*=.11, *p*<.001), and higher frustration at 48 months (*r*=.11, *p*<.001) (Fig. 2; Table S7).

### Which temperament measures, at which ages, best predict CD symptoms at age 7?

Using all 39 predictors, the random forest procedure selected a model with 9 predictors at each split, which explained 1% of the variance in CD symptoms (*R*^*2*^=0.01, *RMSE*=0.90). RFE procedures did not improve the explained variance in CD symptoms (Table S6). Thus, we retained all 39 predictors. Variable importance rankings from the full model are shown in Fig. 1b. The top 10 predictors across the full and RFE models are as follows, with correlation coefficients shown to index directionality: lower attention at 24 months (*r*=-.07, *p*<.05), lower happiness at 24 months (*r*=-.06, *p*=.07), lower fear to new/strange at 24 months (*r*=-.07, *p*<.05), lower fear to new/strange at 48 months (*r*=-.07, *p*<.05), and higher activity at 35 months (*r*=.10, *p*<.01). There were no linear relationships with CD symptoms for responsiveness to persons at 24 (*r*=.00, *p*=.94) and 6 months (*r*=-.02, *p*=.58), happiness at 15 months (*r*=.02, *p*=.47), irritability at 24 months (*r*=.02, *p*=.56), and responsiveness to examiner at 35 months (*r*=.05, *p*=.12), (Fig. 2; Table S7).

### Which temperament predictors are specific to CU traits?

Results of *post hoc* path models using domain scores computed across all 39 features showed that lower affiliation scores were uniquely related to higher CU traits (*β*=-0.14, *p*<.001), but not to CD or ADHD symptoms (Table 3**)**. In addition, lower self-regulation (*β*=-0.21, *p*<.001) and higher activity (*β* = 0.17, *p<.*001) domain scores were uniquely related to more ADHD symptoms, but not CU traits **(**Table 3). In RF models predicting residualized CU traits regressing out conduct disorder symptoms, the top 10 predictors were largely consistent with those of original models (Figure S1). That is, 70% (*n* = 7) of the top predictors overlapped with the original top 10 predictors of CU traits at age 7 (Figure S1).

**Table 3:** Post Hoc Regression Models Testing the Specificity of Domain-Level Predictors of CU Traits, CD Symptoms, and ADHD Symptoms

	CU traits			CD symptoms			ADHD symptoms		
	B (SE)	β	*p*	B (SE)	β	*p*	B (SE)	β	*p*
Activity	− 0.01 (0.01)	− 0.03	0.35	0.01 (0.01)	0.03	0.41	0.06 (0.01)	0.17	< 0.001
Affiliation	− 0.02 (0.01)	− 0.14	< 0.001	0.01 (0.01)	0.05	0.36	0.00 (0.01)	0.01	0.89
Fear	− 0.01 (0.01)	− 0.04	0.23	− 0.02 (0.01)	− 0.06	0.13	− 0.01 (0.01)	− 0.04	0.29
Negative emotionality	− 0.01 (0.02)	− 0.01	0.74	− 0.02 (0.02)	− 0.06	0.19	− 0.01 (0.02)	− 0.03	0.48
Self-regulation	0.01 (0.02)	0.02	0.66	− 0.03 (0.02)	− 0.12	0.04	− 0.07 (0.01)	− 0.21	< 0.001
CU traits				0.27 (0.03)	0.27	< 0.001	0.41 (0.03)	0.41	< 0.001
CD symptoms	0.23 (0.03)	0.23	< 0.001				0.17 (0.03)	0.17	< 0.001
ADHD symptoms	0.43 (0.03)	0.40	< 0.001	0.22 (0.04)	0.22	< 0.001			

See Table 2 for a summary of the predictors included in each cross-measure construct domain

## Discussion

We used ML to identify prospective temperament predictors of CU traits and CD symptoms and test the STAR model. Models explained only 2% of the variance in CU traits and 1% of the variance in CD symptoms, suggesting that these temperament constructs, at least when assessed at the single-item level, are limited in their capacity to predict outcomes at age 7. In support of the STAR model predictions, our ML algorithm identified 4 of the top 10 most important predictors of CU traits at age 7 as constructs relevant to conceptualizations of affiliation (i.e., low positive affect/happiness at 24, 35, and 48 months and low responsiveness to examiner at 24 months). Evidence that low positive affect/happiness at two separate assessments in early childhood prospectively relate to CU traits mirrors prior evidence linking behavioral markers of low affiliation in toddlerhood and early childhood with CU traits [13, 17]. Moreover, in *post hoc* regression models, lower affiliation domain scores derived from the full 39 feature set were specifically related to higher CU traits, but not CD or ADHD symptoms. However, in the context of the small magnitude of variance explained, our results provide only modest support for the importance of affiliation as a unique precursor to the development of CU traits.

One possible explanation for the minimal variance explained may be that observed temperament measures only provide weak prediction of CU traits reported several years later. However, given the breadth of evidence prospectively linking early temperament to psychopathology into adulthood [50, 51], including measures of behavioral fear and inhibition, it is more likely that the predictors included in this study suffered from limited signal. That is, our observed measures of affiliation may not have provided the construct coverage needed to strongly predict future CU traits. Specifically, positive affect and responsiveness in early childhood may be weakly predictive of CU traits when not measured in conjunction with other indicators of affiliation, including social imitation, cooperativeness, and enthusiasm specific to social interactions [5, 16–17]. Additionally, these early temperament measures may provide stronger prediction when combined with measures of parent behavior, as prior work has shown low social affiliation at age 2 specifically predicted CU traits at age 3 when children also experienced low parental positivity [17]. Further, observational measures are inherently subjective, potentially obscuring true associations with future CU traits.

Importantly, in contrast to the hypotheses of the STAR model, no predictors indexing low fear emerged in ML models among the top predictors of CU traits, nor did low scores on the fear domain across all items from the full 39 feature set relate to CU traits in regression models. This finding contrasts with the results of prior studies that have emphasized diminished fear as a risk factor for CU traits [9–11, 24] or that have linked fearlessness to the broader psychopathy phenotype [50]. One possibility is that the profile of temperament risk factors most predictive of CU traits may vary depending on the subtype of CU traits examined (e.g., primary vs. secondary CU traits), which is an avenue for future research [12]. Alternatively, our brief measure of fearlessness (i.e., ratings of child’s fear reactivity to a scary mask) may not have fully captured the fear construct, which encompasses fear to both social (i.e., anger, distress of others) and non-social (i.e., danger, punishment) cues.

Another possibility for our failure to replicate links between low fear and CU traits is that we used a naturalistic context that was designed to elicit fear in the presence of *novelty*, which have different prediction of CU traits compared to low fear elicited in the presence of socially threatening stimuli (e.g., angry or fearful faces). Fear to novelty may be less predictive of CU traits relative to affiliation in contexts that are not designed to induce negative social interactions. Finally, evidence suggests that associations between CU traits and fear change across development, with prior work showing positive associations between fear at 15 months and CU traits in first grade [24] but negative associations between fear at age 3 and CU traits in adulthood [50]. Thus, the developmental timing and observational context in which our behavioral indicators were measured may have resulted in weaker prediction of CU traits.

More broadly, our reliance on brief, often single-item ratings of fear and affiliation may not have captured richness in the two constructs at a granular level, including eye contact, imitation, affection (i.e., affiliation) or responsiveness to negative emotion cues (i.e., fear), undermining our predictive power in relation to later CU traits [5, 15–16]. These items provide a parsimonious yet potentially only partial representation of our higher-order constructs of interest. Indeed, responsiveness was predominantly operationalized as friendliness and interest in persons present, which may not be as relevant for predicting CU traits as other measures capturing *enjoyment* of social interactions that have been included in prior studies, such as physical and verbal affection and smiling [18], eye contact, and cooperation [13]. Our findings inform the STAR model to show that measurement matters, potentially indicating substantial variation in capacity for behavioral observations of fear and affiliation to strongly predict risk for CU traits.

It is also important to consider that possibility that the predictive relationship between STAR features and CU traits attenuates over time, with other factors playing a stronger role as the child develops. For example, behavioral observations of affiliation and fear may be insufficient to predict CU traits several years later without integrating other indicators of risk, including biological (neurobiology, physiology, genetics) and environmental factors (e.g., parenting, peer influences). Indeed, CU traits are known to develop in the context of both environmental risk factors and heritable temperament risk factors [5, 11, 18]. Thus, incorporating indicators of the caregiving environment into models (e.g., parental warmth, harsh discipline) may better explain variance in CU traits in middle childhood and preadolescence. Additionally, each predictor in our dataset was treated as a static risk factor, which precluded the examination of how intra-individual changes in our predictors over time may influence prediction. Thus, it is possible that the growth trajectory of fear and affiliation over time may be more predictive of CU traits than momentary levels of fear and affiliation at a single timepoint. Finally, we must also consider the possibility that we may not have sufficiently strong evidence for the STAR model. Failure to consistently detect low fear and low affiliation as predictors of CU traits may point to the need for caution in assertions that low fear and low affiliation are *necessary* determinants of CU traits.

We also found that low task persistence at 35 and 48 months, lower attention at 24 months, and greater activity at 35 and 48 months were in the top 10 predictors of CU traits at age 7. Persistence is the capacity to sustain effort towards a task-oriented goal, making it a critical component of self-regulation and effortful control [52]. One possibility is that children at risk for CU traits could be identified through an uncaring attitude towards important tasks or others’ expectations, which could manifest as lower scores on persistence [53]. Likewise, low persistence and attention (indices of effortful control) [54], alongside low affiliation, could serve as developmental approximations of disinhibition and meanness, phenotypes central to the triarchic model of psychopathic traits [55]. Finally, although studies directly examining the role of activity in the development of CU traits are limited, activity level could reflect approach behaviors and sensation-seeking [56], which have previously been linked to CU traits [57].

However, a more parsimonious explanation for these findings is that low task persistence, low attention, and activity are all indicators of ADHD, which is known to co-occur with CU traits. That is, the emergence of these features in our top 10 predictors could simply reflect the correlation of CU traits and ADHD symptoms. Indeed, in *post hoc* regression models, there was no association between the domain scores for self-regulation and activity with CU traits, but both were uniquely related to higher ADHD symptoms. Self-regulation may be a stronger predictor of CU traits when interacting with affiliation (e.g., low positive affect, low responsiveness to examiner), as exemplified in the random forest decision tree **(Figure ****S2****)**, than when considered in isolation, as reflected in *post hoc* regression models. These findings suggest the importance of considering multiple measures of temperament when evaluating risk for CU traits, which may be crucial for differential diagnosis and transdiagnostic treatments that may target shared temperament mechanisms.

In the CD model, fear (to new/strange at 24 and 48 months) and affiliation (happiness at 15 and 24 months, responsiveness to examiner at 35 months, and responsiveness to persons at 6 and 24 months) were top predictors. These findings undermine an argument for the specificity of the STAR dimensions in relation to risk for CU traits. However, while these features emerged as top predictors in the RF models, neither fear nor affiliation domain scores predicted CD symptoms in *post hoc* regression models, whereas we did find evidence for specificity between affiliation and the prediction of CU traits. An explanation for this finding is that CD includes a much more heterogenous set of symptoms, including aggression, rule-breaking, norm violations, and delinquency, representing a broader phenotype than CU traits, which captures a narrower set of affective and uncaring features, including an absence of remorse or concern for others’ feelings and/or important tasks [1]. Importantly, endogenous pathways of risk (i.e., temperamental susceptibility) are known to interact with exogenous factors (e.g., socioeconomic status, household instability, maternal sensitivity) [58] to exacerbate or buffer risk for CD, and environmental factors were not included in the current study.

Our results should be considered alongside several important limitations. First, we used single-item indicators to isolate construct-specific effects on CU traits, separating previously combined affect items into fear and affiliation to preserve domain validity and leverage RF feature importance. However, this approach limited construct coverage and did not distinguish social from nonsocial positive affect, which constrains the operationalization of affiliation. Second, our measures of fear and affiliation were sometimes confounded with each other (e.g., ratings for responsiveness to examiner). That is, a child who scored low on responsiveness could have been low on affiliation or high on fearfulness. Third, we designated a priori constructs to categorize our temperament dimensions, with fear as a distinct construct to negative emotionality. However, prior conceptualizations of negative emotionality include fear, in addition to frustration and irritability [56]. Future studies need more comprehensive, distinct assessments of fear and affiliation. Fourth, we lacked assessments of every predictor at all five time points, undermining our ability to fully test whether temperament predictors collected earlier in development (e.g., 6 and 15 months) were more important for predicting CU traits than those same predictors when measured later in development (e.g., 35 and 48 months). Fifth, our training and testing samples were drawn from the same study population, which included predominantly low-income families from rural regions, limiting the generalizability of our findings. Although machine learning algorithms are designed to optimize generalizability by testing the same model on unseen data, our socioeconomically homogenous sample introduces the potential for sample bias. Future studies are needed to evaluate the replicability of our findings in a more socioeconomically diverse sample. We also relied on a population-based sample, with lower proportions of children with severe CU traits and DBD symptoms than would be found in clinic-referred populations. Further, sex- and subtype-specific (e.g., primary vs. secondary CU traits) effects were not probed, which may have resulted in variations in the set of top predictors. Sixth, our feature set was limited to observed behavioral constructs, precluding the exploration of multimodal prediction. Future studies that integrate environmental, neurobiological, and genetic markers of risk are needed. Finally, ML prediction does not imply causal risk factors for CU traits and CD symptoms, and more work is needed to establish mechanisms driving associations between these temperament constructs and later symptoms. Importantly, thorough external validation of findings is needed given the ethical challenges imposed by data-driven prediction of psychopathology, including the risk for algorithmic biases that reify ethnoracial disparities and reinforce stigma despite variable prediction accuracy [59].

In sum, we used RF models to test which measures of temperament, at which ages, were most important for predicting CU traits and CD in middle childhood. Our models explained only 2% of the variance in CU traits and 1% of the variance in CD symptoms. Although predictor variables were assessed through observational assessments, sometimes nearly 7 years before parent ratings of CU traits and CD symptoms, our model suggests overall that these features alone are weak predictors. Moreover, we did not find consistent evidence that the STAR model dimensions outperformed other dimensions of temperament or demonstrated specificity in predicting CU traits. Nevertheless, we provide preliminary evidence that a temperament style characterized by a reduced tendency to respond positively to social interactions may be particularly relevant for characterizing risk for CU traits. Our findings support the future use of machine learning to inform clinical assessment, guide longitudinal research, and advance precision treatments for an important subgroup of children at risk for particularly harmful lifespan trajectories of antisocial behavior.

## Supplementary Information

Below is the link to the electronic supplementary material.

Supplementary Material 1(DOCX 410 KB)

Supplementary Material 2(XLSX 16.8 KB)

## Data Availability

Data are available from the authors upon request.
