# Supplementary material for: Identifying prospective temperament predictors of callous-unemotional traits using machine learning
Source: Eur Child Adolesc Psychiatry. 2026 Mar 19;35(7):2167–78. doi: 10.1007/s00787-026-03012-8 (PMC13378790; doi:10.1007/s00787-026-03012-8)
Supplement: Supplementary file 1 — Supplementary Material 1(DOCX 410 KB) [file 787_2026_3012_MOESM1_ESM.docx]

**Supplemental Materials**

**Identifying Prospective Temperament Predictors of Callous-Unemotional Traits Using Machine Learning**

European Child & Adolescent Psychiatry

Alexis Broussard, MA, Sarah C. Vogel, PhD, Patrick K. Goh, PhD, Emily R. Perkins, PhD, Yael Paz, PhD, Nicole Huth, BA. Anthony J. Rosellini, PhD, William R. Mills-Koonce, PhD, Michael T. Willoughby, PhD, ^Nicholas J. Wagner, PhD, ^Rebecca Waller, PhD.

Dr. Rebecca Waller is with the Department of Psychology at the University of Pennsylvania, Philadelphia, Pennsylvania. Dr. Nicholas J. Wagner is with the Department of Brain and Psychological Science at Boston University, Boston, Massachusetts.

Correspondence: Dr. Rebecca Waller, Department of Psychology, Stephen A. Levin Building, 425 South University Avenue, Philadelphia, PA, 19104; email: rwaller@sas.upenn.edu; phone: 215-898-9394. Dr. Nicholas Wagner, Department of Psychological and Brain Sciences, Boston University, 64 Cummington Mall, Boston, MA, 02215; Email: njwagner@bu.edu. Phone: 6​1​7​-3​5​3​-7370 (^joint senior authors)

Table of Contents

[Table S1. Associations between exclusion status, sample demographics, and outcomes 2](#_Toc216901829)

[Table S2. Pearson’s correlations between ratings from two visits at one timepoint 3](#_Toc216901830)

[Table S3. Data dictionary of study variables 4](#_Toc216901831)

[Table S4. Confirmatory factor analyses of domain-level constructs included in post-hoc regression models predicting CU traits, CD, and ADHD 0](#_Toc216901832)

[Table S5. Descriptive statistics for CU traits, CD symptoms, and ADHD symptoms 0](#_Toc216901833)

[Table S6. Model results across full and RFE models and t-tests reporting no significant differences in performance 1](#_Toc216901834)

[Table S7. Pairwise correlations between predictors and outcomes 2](#_Toc216901835)

[Table S8. Variable importance values from full and RFE models predicting CU traits 0](#_Toc216901836)

[Table S9. Variable importance values from full and RFE models predicting CD symptoms 1](#_Toc216901837)

[Figure S1. Top predictors of original CU traits scores and residualized CU traits scores 2](#_Toc216901838)

[Figure S2. Example of a decision tree from the random forest model predicting CU traits. 3](#_Toc216901839)

# Table S1. Associations between exclusion status, sample demographics, and outcomes

|  | ***X^2^ or t*** | ***p*** |
| --- | --- | --- |
| **Race** | .08 | .78 |
| **Sex** | .24 | .62 |
| **Site** | 2.02 | .16 |
| **Income-to-needs-ratio** | -.79 | .43 |
| **CU Traits** | 1.92 | .06 |
| **CD symptoms** | .95 | .35 |
| **ADHD symptoms** | .41 | .35 |

***Note.*** Sites included North Carolina and Pennsylvania.

# Table S2. Pearson’s correlations between ratings from two visits at one timepoint

|  | **Ages** | | | | | |
| --- | --- | --- | --- | --- | --- | --- |
|  | **6 months (IBR)** | | **24 months (IBR)** | | **35 months (OCTS)** | |
| **Variable Pairs** | ***r*** | ***p*** | ***r*** | ***p*** | ***r*** | ***p*** |
| Attention (Visit A – B) | .53 | < .001 | .59 | < .001 |  |  |
| Gross movement/Activity (Visit A – B) | .60 | < .001 | .59 | < .001 | .58 | < .001 |
| Irritability (Visit A – B) | .35 | < .001 | .50 | < .001 |  |  |
| Responsiveness to persons (Visit A – B) | .51 | < .001 | .58 | < .001 |  |  |
| Responsiveness to examiner (Visit A – B) | .53 | < .001 | .67 | < .001 | .60 | < .001 |
| Responsiveness to caregiver (Visit A – B) | .58 | < .001 | .56 | < .001 |  |  |
| Reaction to new/strange (Visit A – B) | .43 | < .001 | .55 | < .001 | .60 | < .001 |
| Happiness/Positive Affect (Visit A – B) | .45 | < .001 | .56 | < .001 | .55 | < .001 |
| Frustration (Visit A – B) |  |  |  |  | .64 | < .001 |
| Persistence (Visit A – B) |  |  |  |  | .61 | < .001 |
| ***Note.*** IBR = Infant Behavior Record. OCTS = Observation of Child Temperament Scale. For 6-, 24-, and 35-month data, there were two visits (A and B) and data was collected from two interviewers at each visit. However, there was only one visit at 15 and 48 months, and data was collected at that visit from two interviewers. Only intercorrelations between ratings where two visits were completed are shown. | | | | | | |

# Table S3. Data dictionary of study variables

| **Measure** | **Variable Name** | **ICC** | **Variable Description** |
| --- | --- | --- | --- |
| Inventory of Callous-Unemotional Traits (ICU)^1^ | cu_var_numeric |  | ICU scores |
| Disruptive Behavior Disorder Rating Scale (DBDRS)^2^ | cd_var_numeric |  | Conduct disorder symptoms |
| Diagnostic and Statistical Manual of Mental Disorders-IV(DSM-IV-TR)^3^ | ADHD_var_numeric |  | ADHD symptoms |
| The Mask Task^4^ | LTrctM_comp_6, _15, _24 |  | Fear reactivity to mask at 6,15,24 months |
| Infant Behavior Record (IBR)^5^ | IBRResp_psn_6, _15, _24 | .54 - .61 | Responsiveness to persons at 6,15,24 months |
|  | IBRResp_exmr_6, _15, _24 | .59 - .82 | Responsiveness to examiner at 6,15,24 months |
|  | IBRResp_cgr_6, _15, _24 | .50 - .72 | Responsiveness to caregiver at 6, 15, 24 months |
|  | IBRrct_new_6, _15, _24 | .53 - .74 | Reaction to new/strange at 6, 15, 24 months |
|  | IBRAttn_6, _15, _24 | .71 - .82 | Attention at 6, 15, 24 months |
|  | IBRGmov_6, _15, _24 | .67 - .74 | Gross movement at 6, 15, 24 months |
|  | IBRIrit_6, _15, _24 | .73 - .79 | Irritability at 6, 15, 24 months |
|  | IBRHapp_6, _15, _24 | .66 - .83 | Happiness at 6, 15, 24 months |
| Observation of Child Temperament Scale (OCTS)^5^ | OCT_new_psn_35, _48 | .68 - .73 | Responsiveness to examiner at 35, 48 months |
|  | OCT_act_35, _48 | .68 - .70 | Activity at 35, 48 months |
|  | OCT_frust_35, _48 | .68 - .80 | Frustration at 35, 48 months |
|  | OCT_posaff_35, _48 | .60 - .70 | Positive affect at 35, 48 months |
|  | OCT_shyfear_35, _48 | .69 - .81 | Fear to new/strange at 35, 48 months |
|  | OCT_tskpers_35, _48 | .71 - .75 | Persistence at 35, 48 months |

***Note.*** ICC = Intraclasss Correlation Coefficient.

# Table S4. Confirmatory factor analyses of domain-level constructs included in post-hoc regression models predicting CU traits, CD symptoms, and ADHD symptoms

|  | **CFI** | **RMSEA** | ***X^2^*** | ***df*** |
| --- | --- | --- | --- | --- |
| **Fear** | .96 | .04 | 42.50*** | 15 |
| **Affiliation** | .94 | .06 | 383.80*** | 83 |
| **Self -Regulation** | .97 | .07 | 23.87*** | 4 |
| **Activity** | .96 | .07 | 20.56*** | 4 |
| **Negative Emotionality** | .97 | .05 | 13.85** | 4 |

***Note.*** ****p* < .001, ***p* < .01, **p* < .05. A unidimensional model was created for each domain-level construct, with all within-domain items loading onto a single domain-level factor. Within-domain items were subjected to CFA with robust maximum likelihood estimation. We allowed for within-timepoint correlations between observed indicators of each domain-level construct to account for the shared variance between indicators measured at the same timepoint. The latent factor fear included 8 items indexing fear reactivity and fear to new/strange across 6, 15, 24, 35, and 48 months. After checking modification indices for the unidimensional fear model, fear reactivity at 15 and 24 months were allowed to correlate with each other, along with fear reactivity at 6 and 15 months, to improve model fit. The latent factor affiliation included 16 items indexing responsiveness to persons, examiner, and caregiver as well as happiness/positive affect across 6, 15, 24, 35, and 48 months. After checking modification indices for the unidimensional affiliation model, responsiveness to examiner at 35 and 48 months were allowed to correlate with each other to improve model fit. The latent factor self-regulation included 5 items indexing attention and persistence across 6, 15, 24, 35, and 48 months. After checking modification indices for the unidimensional self-regulation model, persistence at 35 and 48 months were allowed to correlate with each other to improve model fit. The latent factor activity included 5 items indexing gross movement/activity across 6, 15, 24, 35, and 48 months. After checking modification indices for the unidimensional activity model, activity at 35 and 48 months were allowed to correlate with each other to improve model fit. The latent factor negative emotionality included 5 items indexing irritability and frustration across 6, 15, 24, 35, and 48 months. After checking modification indices for the unidimensional negative emotionality model, frustration at 35 and 48 months were allowed to correlate with each other to improve model fit.

# Table S5. Descriptive statistics for CU traits, CD symptoms, and ADHD symptoms

|  | **Mean** | **SD** | **Median** | **Range** | **Skew** | **Kurtosis** | $\boldsymbol{\alpha}$ |
| --- | --- | --- | --- | --- | --- | --- | --- |
| **CU traits** | 16.61 | 9.77 | 15 | 0 - 53 | .60 | -.13 | .71 |
| **CD symptoms** | .23 | .90 | 0 | 0 - 9 | 5.66 | 39.17 | .92 |
| **ADHD symptoms** | 15.54 | 11.67 | 12.67 | 0 - 57 | 1.01 | 0.73 | .95 |

# Table S6. Model results across full and RFE models and t-tests reporting no significant differences in performance

| **CU Traits** | | | | | | | | | | | | |
| --- | --- | --- | --- | --- | --- | --- | --- | --- | --- | --- | --- | --- |
|  | **Training Data** | | | | **Testing Data** | | | | **Model Differences** | | | |
|  | Full Model (39 features) | | Post-RFE Model (37 features) | | Full Model (39 features) | | Post-RFE Model (37 features) | | *t* | | | *p* |
| **mtry** | 3 | | 2 | |  | |  | |  | | |  |
| **Rsquared** | .06 | | .07 | | .02 | | .02 | | -.75 | | | .46 |
| **RMSE** | 9.65 | | 9.63 | | 9.20 | | 9.17 | | .17 | | | .86 |
| **CD Symptoms** | | | | | | | | | | | | |
|  | | **Training Data** | | | | **Testing Data** | | | | **Model Differences** | | |
|  |  | Full Model (39 features) | | Post-RFE Model (4 features) | | Full Model (39 features) | | Post-RFE Model (4 features) | | *t* | *p* | |
| **mtry** | | 9 | | 4 | |  | |  | |  |  | |
| **Rsquared** | | .03 | | .09 | | .01 | | .04 | | -1.89 | .07 | |
| **RMSE** | | .89 | | .88 | | .90 | | .91 | | .02 | .98 | |

***Note.*** ****p* < .001, ***p* < .01, **p* < .05.

# Table S7. Pairwise correlations between predictors and outcomes

Separate excel file attachment.

***Note.*** ****p* < .001, ***p* < .01, **p* < .05. See **Table S3** for data dictionary of study variables.

# Table S8. Variable importance values from full and RFE models predicting CU traits

| **Full Model (39 features)** | | **RFE (37 features)** | | **Post-RFE Model (37 features)** | |
| --- | --- | --- | --- | --- | --- |
| **Features** | **Importance** | **Features** | **Importance** | **Features** | **Importance** |
| OCT_posaff_48 | 100 | OCT_posaff_48 | 8.64083887 | OCT_posaff_48 | 100 |
| OCT_new_psn_48 | 61.48801712 | OCT_frust_48 | 5.82349715 | OCT_tskpers_48 | 92.017364 |
| IBRResp_exmr_24 | 61.38181659 | IBRAttn_24 | 4.68545164 | OCT_frust_48 | 88.3117005 |
| OCT_tskpers_48 | 58.23848663 | IBRHapp_24 | 3.92428362 | OCT_new_psn_48 | 81.2005214 |
| OCT_tskpers_35 | 54.99747519 | OCT_tskpers_48 | 3.78475218 | IBRHapp_24 | 79.552511 |
| IBRResp_psn_15 | 52.71650433 | IBRResp_psn_24 | 3.73810367 | OCT_tskpers_35 | 73.8954521 |
| OCT_frust_48 | 52.11967325 | OCT_tskpers_35 | 3.69494099 | IBRIrit_24 | 72.1362038 |
| IBRResp_psn_24 | 52.10117467 | OCT_act_48 | 3.55406402 | IBRGmov_24 | 7.1613714 |
| IBRAttn_15 | 49.90042914 | OCT_posaff_35 | 3.32054358 | IBRResp_exmr_24 | 67.6526488 |
| IBRHapp_24 | 49.75946378 | OCT_shyfear_48 | 3.19891704 | OCT_posaff_35 | 65.937035 |
| OCT_act_35 | 46.23485874 | IBRIrit_24 | 3.14225688 | IBRAttn_24 | 62.1074801 |
| IBRAttn_24 | 45.54114628 | OCT_new_psn_48 | 3.01435395 | OCT_act_48 | 59.0078614 |
| OCT_posaff_35 | 45.28035201 | OCT_act_35 | 2.94717306 | OCT_act_35 | 55.5857195 |
| OCT_act_48 | 39.19626145 | IBRResp_exmr_24 | 2.6262397 | IBRResp_psn_24 | 54.2783599 |
| OCT_shyfear_48 | 36.57189149 | IBRGmov_24 | 2.56543751 | IBRHapp_15 | 51.2260568 |
| IBRResp_psn_6 | 36.01125441 | IBRResp_psn_15 | 2.19739207 | IBRResp_psn_15 | 49.8646674 |
| IBRGmov_15 | 35.41736578 | IBRrct_new_24 | 2.00138418 | IBRIrit_15 | 46.2080487 |
| IBRrct_new_6 | 32.7160685 | IBRrct_new_6 | 1.86448492 | IBRResp_psn_6 | 43.035897 |
| IBRrct_new_24 | 32.39181218 | IBRGmov_15 | 1.80016661 | IBRrct_new_6 | 42.7009972 |
| IBRIrit_24 | 3.79011606 | IBRResp_exmr_15 | 1.79533541 | OCT_new_psn_35 | 42.3943369 |
| OCT_new_psn_35 | 28.06336775 | IBRHapp_6 | 1.78956739 | IBRAttn_15 | 41.6122544 |
| IBRResp_cgr_24 | 27.1794235 | OCT_new_psn_35 | 1.74650102 | OCT_frust_35 | 39.2907323 |
| IBRHapp_15 | 26.43435803 | IBRAttn_6 | 1.69878942 | IBRrct_new_15 | 39.2852642 |
| LTrctM_comp_6 | 24.74923131 | IBRResp_cgr_15 | 1.65017028 | IBRResp_exmr_15 | 39.1248223 |
| OCT_shyfear_35 | 23.87707073 | IBRGmov_6 | 1.62159393 | IBRResp_cgr_15 | 38.265796 |
| IBRGmov_6 | 23.71075985 | IBRAttn_15 | 1.60937075 | IBRAttn_6 | 35.2085195 |
| IBRrct_new_15 | 23.53091645 | IBRHapp_15 | 1.44860441 | IBRrct_new_24 | 34.6778598 |
| IBRResp_cgr_15 | 23.210404 | IBRIrit_15 | 1.44018737 | IBRResp_exmr_6 | 32.3561777 |
| OCT_frust_35 | 22.55101646 | LTrctM_comp_6 | 1.43242101 | IBRGmov_15 | 26.5558402 |
| IBRResp_exmr_6 | 21.92030911 | IBRResp_exmr_6 | 1.36485309 | IBRResp_cgr_6 | 25.5463968 |
| IBRHapp_6 | 18.49833325 | IBRResp_psn_6 | 1.21085132 | LTrctM_comp_6 | 23.4682958 |
| IBRGmov_24 | 15.85856868 | IBRrct_new_15 | 1.18206371 | OCT_shyfear_48 | 21.3741128 |
| IBRIrit_6 | 14.89158061 | OCT_frust_35 | .82231828 | IBRHapp_6 | 2.4892386 |
| IBRResp_exmr_15 | 14.58538857 | OCT_shyfear_35 | .73419926 | IBRIrit_6 | 16.821555 |
| IBRAttn_6 | 13.79475102 | IBRIrit_6 | .62216673 | LTrctM_comp_24 | 1.854842 |
| LTrctM_comp_15 | 12.16775165 | LTrctM_comp_24 | .48890288 | IBRGmov_6 | 8.00946289 |
| LTrctM_comp_24 | 8.057686741 | IBRResp_cgr_6 | .21542195 | OCT_shyfear_35 | 0 |
| IBRIrit_15 | 6.96736728 |  |  |  |  |
| IBRResp_cgr_6 | 0 |  |  |  |  |

***Note.*** See **Table S3** for data dictionary of study variables.

# Table S9. Variable importance values from full and RFE models predicting CD symptoms

***Note.*** See **Table S3** for data dictionary of study variables.

| **Full Model (39 features)** | | **RFE (4 features)** | | **Post-RFE Model (4 features)** | |
| --- | --- | --- | --- | --- | --- |
| **Features** | **Importance** | **Features** | **Importance** | **Features** | **Importance** |
| IBRAttn_24 | 100 | IBRAttn_24 | 8.79525489 | IBRrct_new_24 | 100 |
| IBRrct_new_24 | 99.80250298 | IBRrct_new_24 | 7.73187688 | IBRAttn_24 | 75.9261373 |
| IBRHapp_24 | 76.10227323 | IBRHapp_24 | 6.73885662 | IBRHapp_24 | 37.9435335 |
| IBRHapp_15 | 75.37794653 | OCT_shyfear_48 | 6.73313361 | OCT_tskpers_48 | 0 |
| OCT_shyfear_48 | 71.13589402 |  |  |  |  |
| IBRResp_psn_6 | 69.42416839 |  |  |  |  |
| OCT_act_35 | 69.16451773 |  |  |  |  |
| IBRIrit_24 | 69.10970223 |  |  |  |  |
| IBRResp_psn_24 | 66.81145626 |  |  |  |  |
| OCT_new_psn_35 | 66.06085582 |  |  |  |  |
| IBRResp_psn_15 | 65.95867221 |  |  |  |  |
| IBRAttn_15 | 65.32707719 |  |  |  |  |
| OCT_new_psn_48 | 64.41919642 |  |  |  |  |
| IBRResp_exmr_24 | 63.18130701 |  |  |  |  |
| OCT_tskpers_35 | 62.47885358 |  |  |  |  |
| OCT_frust_35 | 57.60143814 |  |  |  |  |
| OCT_posaff_48 | 54.59704681 |  |  |  |  |
| IBRGmov_6 | 54.28452395 |  |  |  |  |
| OCT_posaff_35 | 51.11114181 |  |  |  |  |
| IBRrct_new_6 | 5.79420393 |  |  |  |  |
| OCT_tskpers_48 | 5.50358319 |  |  |  |  |
| OCT_act_48 | 49.66692343 |  |  |  |  |
| IBRResp_exmr_6 | 49.59493025 |  |  |  |  |
| OCT_shyfear_35 | 49.54759255 |  |  |  |  |
| IBRAttn_6 | 48.04721867 |  |  |  |  |
| IBRIrit_6 | 47.72815131 |  |  |  |  |
| IBRrct_new_15 | 46.94522376 |  |  |  |  |
| IBRHapp_6 | 45.5092704 |  |  |  |  |
| IBRGmov_15 | 43.57413075 |  |  |  |  |
| IBRResp_cgr_6 | 32.3371485 |  |  |  |  |
| OCT_frust_48 | 3.60497743 |  |  |  |  |
| LTrctM_comp_6 | 3.43087107 |  |  |  |  |
| IBRGmov_24 | 26.75226359 |  |  |  |  |
| IBRResp_cgr_15 | 23.95207587 |  |  |  |  |
| IBRIrit_15 | 2.45950084 |  |  |  |  |
| LTrctM_comp_24 | 19.74918076 |  |  |  |  |
| IBRResp_cgr_24 | 16.71864689 |  |  |  |  |
| LTrctM_comp_15 | 11.89053746 |  |  |  |  |
| IBRResp_exmr_15 | 0 |  |  |  |  |
|  |  |  |  |  |  |
|  |  |  |  |  |  |
|  |  |  |  |  |  |
|  |  |  |  |  |  |
|  |  |  |  |  |  |
|  |  |  |  |  |  |

# Figure S1. Top predictors of original CU traits scores and residualized CU traits scores

***
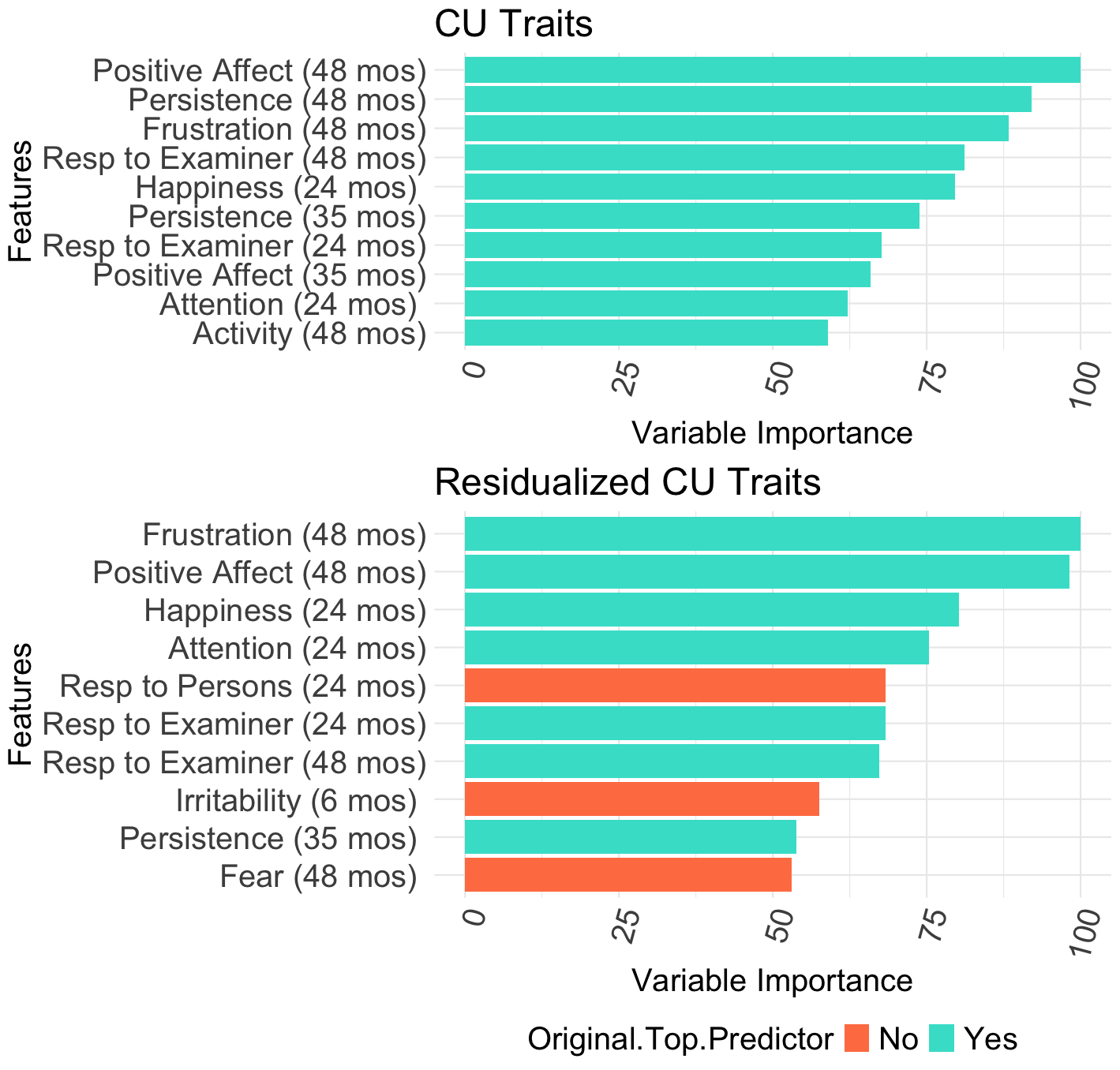
Note.*** Residualized CU traits scores were created by regressing out the variance explained by conduct disorder symptoms. The random forest algorithm used all 39 features to predict residualized CU traits scores, explaining 1% of the variance in the outcome. Figure S1b presents the top 10 predictors of the residualized outcome; 70% (N=7) of the predictors overlap with the original top 10 predictors of CU traits at age 7 (presented in Figure S1a). Only 30% (N=3) are new predictors.

b.

# Figure S2. Example of a decision tree from the random forest model predicting CU traits.


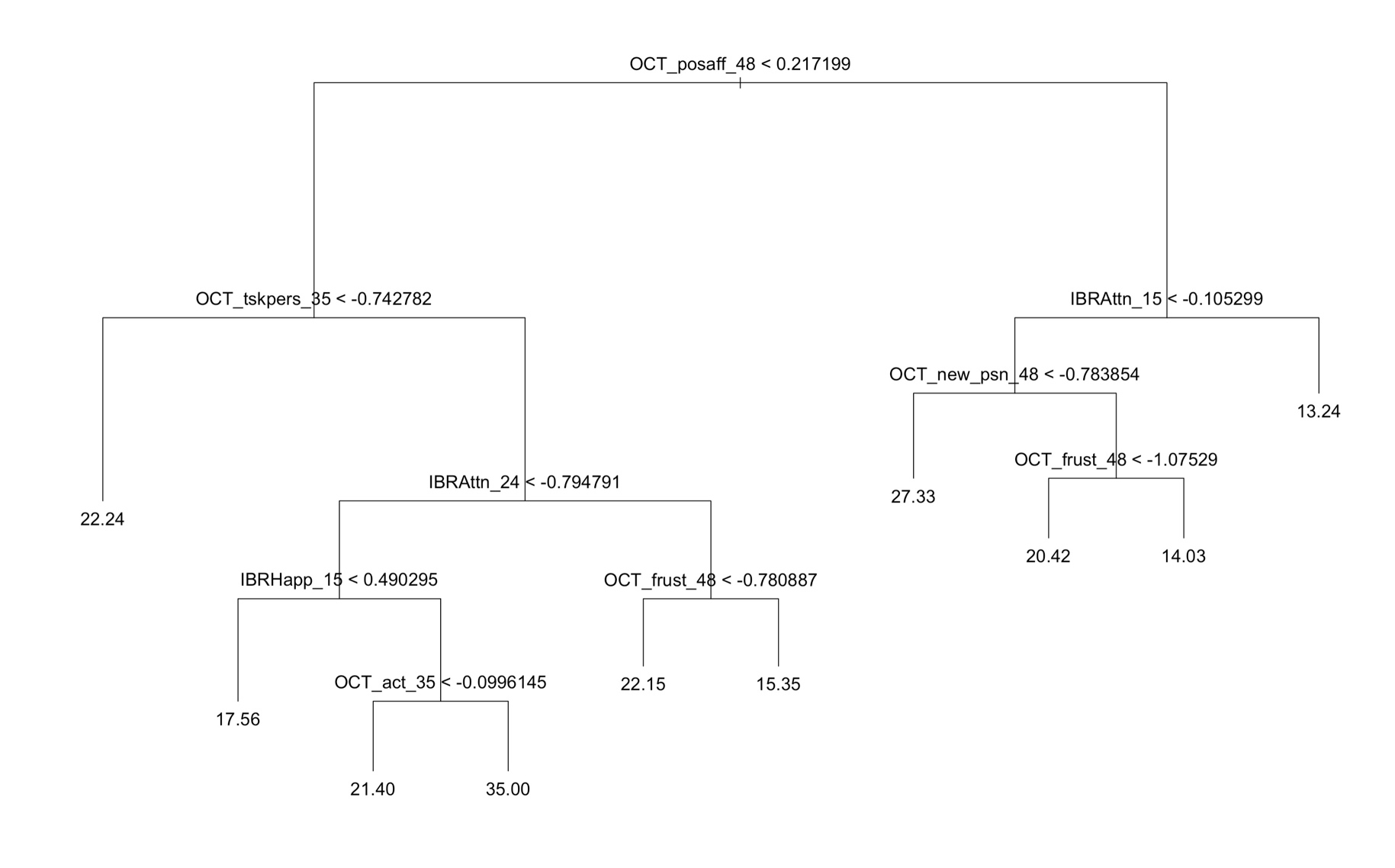


***Note.*** This representative tree models the continuous outcome, CU traits, using the 20 top features and was extracted from a single random forest tree using the R “tree” package.^6^  Each node contains the feature selected for splitting at that node and the value on which it was split represented by a mathematical condition. The cases split to the left daughter node are those for which the condition was met; those in the right node are those for which the condition was not met. The numeric values displayed at each terminal node are the mean values of the outcome variable for participants residing in that terminal node

**Supplemental References**

1. Frick PJ. Inventory of Callous–Unemotional Traits. Unpublished rating scale: University of

New Orleans; 2004.

2. Pelham WE, Gnagy EM, Greenslade KE, Milich R. Teacher ratings of DSM-III-R symptoms

for the disruptive behavior disorders. *J Am Acad Child Adolesc Psychiatry*. 1992;31(2):210-218. doi:10.1097/00004583-199203000-00006

3. American Psychiatric Association. Diagnostic and Statistical Manual of Mental Disorders. 4th ed, text rev. Washington, DC: American Psychiatric Association; 2000.

4. Planalp EM, Van Hulle C, Gagne JR, Goldsmith HH. The infant version of the laboratory temperament assessment battery (Lab-TAB): measurement properties and implications for concepts of temperament. *Front Psychol*. 2017;8:846. doi:10.3389/fpsyg.2017.00846

5. Bayley N. Bayley scales of infant development: manual. New York, NY: The Psychological

Corporation; 1969: 1-178.

6. Ripley B. tree: classification and regression trees. Published online 2023:1.0-44.

doi:1.32614/CRAN.package.tree
